# Compiling Magnetosheath Statistical Data Sets Under Specific Solar Wind Conditions: Lessons Learnt From the Dayside Kinetic Southward IMF GEM Challenge

**DOI:** 10.1029/2020EA001095

**Published:** 2020-05-30

**Authors:** A. P. Dimmock, H. Hietala, Y. Zou

**Affiliations:** ^1^ Swedish Institute of Space Physics Uppsala Sweden; ^2^ Blackett Laboratory Imperial College London London UK; ^3^ Department of Physics and Astronomy University of Turku Turku Finland; ^4^ Department of Earth, Planetary, and Space Sciences University of California Los Angeles CA USA; ^5^ Department of Space Science The University of Alabama in Huntsville Huntsville AL USA

**Keywords:** magnetosheath, statistical analysis, southward IMF, magnetosphere, space weather

## Abstract

The Geospace Environmental Modelling (GEM) community offers a framework for collaborations between modelers, observers, and theoreticians in the form of regular challenges. In many cases, these challenges involve model‐data comparisons to provide wider context to observations or validate model results. To perform meaningful comparisons, a statistical approach is often adopted, which requires the extraction of a large number of measurements from a specific region. However, in complex regions such as the magnetosheath, compiling these data can be difficult. Here, we provide the statistical context of compiling statistical data for the southward IMF GEM challenge initiated by the “Dayside Kinetic Processes in Global Solar Wind‐Magnetosphere Interaction” focus group. It is shown that matching very specific upstream conditions can severely impact the statistical data if limits are imposed on several solar wind parameters. We suggest that future studies that wish to compare simulations and/or single events to statistical data should carefully consider at an early stage the availability of data in context with the upstream criteria. We also demonstrate the importance of how specific IMF conditions are defined, the chosen spacecraft, the region of interest, and how regions are identified automatically. The lessons learnt in this study are of wide context to many future studies as well as GEM challenges. The results also highlight the issue where a global statistical perspective has to be balanced with its relevance to more‐extreme, less‐frequent individual events, which is typically the case in the field of space weather.

## Key Points

- We provide technical guidelines for compiling large‐scale magnetosheath statistical data
- The availability of data for specific simulation/event conditions should be considered in the early planning stages of future studies
- The limits and manner in which solar wind criteria are defined play a major role in compiling statistical datasets

## 1 Introduction

The Sun continually emits a radial stream of charged particles known as the solar wind, which propagates through interplanetary space. The solar wind plasma is often interrupted by obstacles such as planets and comets, in which complex and varied interactions take place. At Earth, the terrestrial magnetic field decelerates the supermagnetosonic solar wind to submagnetosonic speeds, and therefore, a fast‐mode standing shock wave is formed—known as the bow shock. Downstream of the bow shock, the plasma is slower, denser, hotter, and more turbulent compared to its upstream counterpart. This complex region is the magnetosheath, and it extends to the magnetopause, which is a discontinuity formed to the first order, under the pressure balance between the dynamic pressure of the solar wind and the magnetic pressure of the inner‐magnetosphere. The complexity of the magnetosheath is increased by the presence of numerous small‐scale structures, which are also coupled to the solar wind properties. Examples of these are high‐speed jets (Hietala et al., 2012; Plaschke et al., 2018), hot flow anomalies (Omidi et al., 2014; Sibeck et al., 2002), the Kelvin Helmholtz instability (Nykyri et al., 2006), flux transfer events (Russell & Elphic, 1979), and numerous wave modes (Chaston et al., 2013). In general, the magnetosheath is a transition layer, acting as a natural interface between the solar wind and the inner‐magnetosphere. Therefore, it plays a fundamental role as its conditions regulate the processes that transfer mass and momentum to the inner‐magnetosphere.

The magnetosheath has been studied actively for many decades using a variety of approaches (Dimmock & Nykyri, 2013; Fairfield, 1976; Luhmann et al., 1986; Song et al., 1994; Zastenker et al., 2002). In general, studies are individual or a few selected case studies (Moore et al., 2016), numerical modeling (Omidi et al., 2010), larger‐scale statistical studies (Dimmock et al., 2015; Soucek et al., 2015), or a synergy of these (Karimabadi et al., 2014). There are benefits and drawbacks to each of these approaches. Case studies allow for an in‐depth and detailed analysis but lack the global context. Models based on magnetohydrodynamics provide a limited global picture of the magnetosheath but, more importantly, lack the effects on the plasma from kinetic processes. Kinetic hybrid models have advanced our knowledge of the magnetosheath (Von Alfthan et al., 2014), but they exclude electron physics, are sometimes limited to 2‐D, and cannot always be matched with the observations. Large‐scale statistical studies provide a global picture from the observations; however, data coverage is not always sufficient, resulting in uncertainty. It is no surprise that recent studies are adopting combinations of these techniques to overcome the individual limitations of each single methodologies.

Given the presently available and ever‐increasing amount of observational data in heliophysics, supplementing interesting case events with statistical observations seems both appealing and straightforward. However, the magnetic and plasma topology of the magnetosphere is very complex, with numerous boundaries and regions that define several subregions. To add further difficulty, the dimensions of these boundaries are sensitively coupled to the upstream solar wind parameter space. For example, the position of the bow shock and magnetopause can differ by several Earth radii depending on the upstream conditions (Shue et al., 1998). As a result of this, collecting statistical data within the magnetosheath and other dynamical regions (e.g., cusps and inner‐magnetosphere) is not a straightforward task. There are, however, techniques that can be applied to overcome these difficulties, and these have been successfully applied in numerous studies (Chaston et al., 2013; Dimmock & Nykyri, 2013; Soucek et al., 2015; Yao et al., 2011). The difficulty associated with statistical investigations is that large amounts of data are required. Fortunately, there are several long‐duration missions (e.g., Cluster, THEMIS, and MMS), which have surveyed these regions for several years (MMS), and even decades (THEMIS and Cluster).

Although the motivation for this report is the recent southward Interplanetary Magnetic Field (IMF) Geospace Environment Modelling (GEM) challenge hosted by the “Dayside Kinetic Processes in Global Solar Wind‐Magnetosphere Interaction” focus group, this manuscript will be of interest to many other studies. In general, southward IMF is the most geoeffective upstream condition due to dayside subsolar reconnection. Understanding the local and global magnetosheath dynamical behavior during these conditions is highly important, both to fundamental and applied aspects of magnetospheric physics. The goals of this challenge were to study a specific southward IMF event using multiple kinetic models and observational data sets. Over the course of the challenge, a series of model‐data and model‐model comparisons were carried out with the overarching goals to (1) advance our understanding of the various kinetic phenomena observed in models and experimental data, (2) better understand the differences between these phenomena across the models and observations, and (3) determine appropriate metrics for validation purposes. Many other studies will share these or similar goals, and therefore, the lessons documented here will be of a wide scope, which reach beyond the GEM community.

Comparing measurements from spacecraft along individual orbital traces with similar trajectories within a model can be useful; however, it is often required that a more large‐scale comparison is made. In this case, a statistical approach is often adopted since large amounts of locally measured data spatially distributed over a wide area can be used to obtain a global picture. Nevertheless, as mentioned above, large‐scale statistical studies of the magnetosheath are challenging, especially for specific upstream solar wind conditions. In this text, the statistical context of this challenge will be described while providing a brief overview of the methods commonly adopted for the statistical study of the magnetosheath. Although we place specific emphasis on the application to this GEM challenge condition, the broader context will be discussed. The application to other regions in the magnetosphere will also be briefly mentioned to demonstrate that the difficulties mentioned are shared across many key regions in the magnetosphere.

## 2 Data

For this specific study, equatorial magnetospheric measurements are composed of THEMIS (Angelopoulos, 2008) observations between 2007 and 2018. Magnetic field data were recorded by the fluxgate magnetometer (Auster et al., 2008), of which we use both the FGS spin (0.33 Hz) and FGL low (4 Hz) data where appropriate. Ion measurements are taken from the Level 2 moments of the ElectroStatic Analyser (ESA) instrument (McFadden et al., 2008) at a cadence of 0.33 Hz. The OMNI database was accessed via the OMNIweb (omniweb.gsfc.nasa.gov) service to obtain upstream solar wind parameters. These data were employed to (1) add upstream context to magnetosheath measurements and (2) evaluate the magnetosheath boundary models. OMNI data are composed of ACE and Wind measurements conducted at the L1 Lagrange point, which are then propagated (King & Papitashvili, 2005) to the bow shock nose location according to the model by Farris and Russell (1994). Thus, they are not measured in situ but are representative of the conditions immediately upstream of the bow shock. Cusp measurements are from Cluster (Balogh et al., 1997) and were obtained via the Cluster science archive (https://www.cosmos.esa.int/web/csa).

## 3 Methods for Automatic Identification of the Magnetosheath

One of the main difficulties in conducting a large‐scale statistical study of the magnetosheath is the fundamental requirement to identify the intervals when a spacecraft occupies this (or any) dynamic region. The source of the problem lies in the fact that boundaries of the magnetosheath are extremely variable and their positions can differ by several Earth radii depending on the prevailing solar wind conditions (Shue et al., 1998; Verigin et al., 2003). In addition, the physical properties of the bow shock are asymmetrical about the Sun‐Earth line (Verigin et al., 2001). In general, this arises due to the contrasting geometry on each flank caused by the the frequent Parker‐spiral IMF orientation. Nonetheless, because of the readily available extensive catalogs of magnetosheath observations, it is no surprise that there are abundant statistical studies of the magnetosheath and its internal processes (e.g., waves, turbulence, and instabilities).

In practice, one of two approaches are adopted. The first approach, which we will call the boundary ID method, is to employ models of the bow shock and magnetopause to determine if a spacecraft is located within the model magnetosheath (Verigin et al., 2006); this is made possible with long‐term solar wind measurements such as OMNI. The second approach is to analyze the in situ field and plasma measurements to build criteria that corresponds to magnetosheath conditions (Chaston et al., 2013), which can then be applied in order to extract intervals of magnetosheath encounters. It is worth noting that both methods are reliant upon in situ measurements in the sense that the majority of boundary models, included those used here, are derived empirically from observations. The main difference between these two methods is that the boundary ID approach can estimate the radial distance across the magnetosheath and therefore the expansion and compression of the region can be accounted for. In general, the boundary ID approach is useful if one needs to consider the location within the magnetosheath. On the other hand, in situ data may be a more accurate way to identify the magnetosheath in close proximity to the boundaries. This can be due to model accuracy, and also because the model boundary represents an average position. This does not account for local processes such as reconnection, waves, and instabilities, which can drive small‐scale perturbations of the boundary.

In this section, the majority of attention will be devoted to the boundary ID method, but the second will be briefly discussed as well. The goal is to advise readers who are considering compiling magnetosheath statistics. This problem has become increasingly relevant with the ever‐increasing sophistication of global simulations, which should be compared against experimental data.

### 3.1 Identification Based on Model Boundaries

The first approach is to determine the boundary locations with respect to the spacecraft position. This method requires access to models of both the inner and outer boundaries of the magnetosheath and upstream solar wind measurements (for inputs to these models). As a result, for all practical purposes, this method is limited to the terrestrial magnetosheath where a solar wind monitor is available. The first step is to determine the position of the magnetopause (*r*_*mp*_) and bow shock (*r*_*bs*_) along the radial spacecraft direction (**R**). This permits the evaluation of a normalized distance (*F*) along the magnetosheath as follows:

(1) $$F=\frac{|R|-r_{mp}}{r_{bs}-r_{mp}}.$$

In this case, the values of *F* correspond to the following regions:

(2) $$F=\left\{\begin{array}{rl} \left[0\to 1\right] & \text{magnetosheath}\\ <0 & \text{magnetosphere}\\ >1 & \text{solar wind}\\ 1 & \text{bow shock}\\ 0 & \text{magnetopause} \end{array}\right..$$

Therefore, the magnetosheath can be identified when *F* lies between 0 and 1. More importantly, the fraction distance (*F*) accounts for the physical variations in shape and size of the magnetosheath and allows the direct comparison of large‐scale statistical observations, which were collected during contrasting upstream conditions. Another benefit is that studies can be performed in various regions within the magnetosheath, such as close to the magnetopause, bow shock, or in the central magnetosheath away from both boundaries.

The transformation from physical to normalized coordinates can be implemented for various coordinate systems such as the Geocentric solar ecliptic (GSE), Geocentric solar magnetospheric (GSM), and the Magnetosheath InterPlanetary Medium (MIPM) frame. In each coordinate frame, typically, the planetary aberration angle (a few degrees) is also corrected for by first rotating into a system in which the *x* axis is antiparallel to the upstream solar wind flow direction; note that the orbital motion is removed from*V*_*y*_. If OMNI data are used, then the orbital motion does not need to be removed since it is already implemented in the OMNI data‐processing pipeline. For more technical details of this frame and multiple examples of its implementation, see Verigin et al. (2006) and Dimmock et al. (2017).

It is important to note that model boundary positions are subject to error and will not always match the true boundary position, resulting in misidentified regions. In practice, this will add data points measured outside of the magnetosheath (e.g., solar wind) to the magnetosheath statistics. However, additional criteria on the in situ data can be added to check for these outliers. For example, for dayside magnetosheath studies, it is reasonable to require that the magnetosheath flow speed is less than its upstream counterpart, that is,*V*_*ms*_/*V*_*sw*_<1. Other criteria can also be applied to remove outliers, such as limiting ion temperature or entropy. However, these conditions should be selected based on the requirements of each investigation. Nevertheless, considering that they are straightforward to implement, they are recommended.

Intervals during highly variable upstream conditions can also be removed. Since this method typically involves averaging data over a given window, this condition can easily be checked. In reality, limits can be placed on the permitted variability (e.g., standard deviation) of the measured parameters within these windows. In past studies (Dimmock et al., 2017) typically 3‐ and 20‐min windows have been used for the magnetosheath and upstream measurements, respectively. Removing cases of significant variability can reduce the inaccuracy of the model position and improve the validity of averages taken during an interval. Unfortunately, these conditions are likely to occur during extreme events such as large storms, when enhanced variability takes place and therefore limits the applicability for these types of driving conditions. Therefore, it may be advisable to implement this by imposing an allowable standard deviation or equivalent metric on each window. It should be noted that each criterion will result in some data loss, and in general, there is a trade‐off between any criteria limits and the required statistical accuracy. This should be balanced by taking into consideration the data availability and required accuracy.

**Figure 1:**
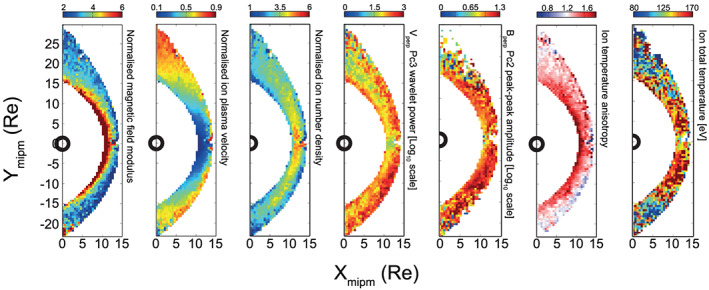
Statistical maps compiled using the automated boundary identification methodology. Shown are various measured and derived plasma and field properties binned in the dayside magnetosheath for the interval January 2008 to December 2015.

Figure 1 shows several examples of the implementation of this methodology using THEMIS data between 2008 and 2015. Each panel corresponds to a different measured or derived quantity, demonstrating that many aspects of solar wind‐magnetosheath coupling can be studied in this manner. It should also be noted that these maps can be reproduced based on subsets corresponding to various upstream solar wind conditions.

The statistical maps shown in Figure 1 were compiled by first evaluating the Verigin et al. (2003) and Shue et al. (1998) bow shock and magnetopause models using 20‐min‐averaged OMNI data. For magnetosheath measurements, a 3‐min window of THEMIS measurements were analyzed to yield one data point, which is typically a mean or median average. These values are then later binned into a 0.5 × 0.5 Re grid based on their cartesian coordinate. The mean of these points within each bin was used to construct the bin value; however, other metrics such as standard deviation and range can easily be applied. The coordinate system used here is the MIPM reference frame, which aims to gather points that were processed by a quasi‐perpendicular (quasi‐parallel) shock geometry on the dusk (dawn) flank. This frame is particularly useful for analyzing the impact on the magnetosheath from the contrasting shock geometries on the dawn and dusk flanks. For more details, see Verigin et al. (2006). Note that these maps can be produced for other coordinate systems such as GSE and GSM.

### 3.2 Identification Based on In Situ Spacecraft Measurements

As mentioned, the plasma properties in the magnetosheath are markedly different to those in the surrounding regions. Compared to the solar wind, the magnetosheath is hotter, denser, slower, and significantly more turbulent. In comparison to the inner‐magnetosphere, the magnetosheath plasma is generally cooler, faster, denser, and more turbulent. Based on the distinction between plasma parameters across these regions, it is possible to identify the magnetosheath based on the position of the spacecraft and the measured plasma and field properties. If simultaneous upstream solar wind conditions are available, then these can also be used to improve accuracy. The point here is that the magnetosheath can be identified even if the boundary locations are unknown. The most difficult aspect of this method is that it requires the derivation of specific criteria, which are defined well enough so that it is possible to identify magnetosheath intervals while allowing for inevitable variability. However, the issue here is that the parameter range of the magnetosheath can be extremely large, making it difficult to define set criteria. To complicate matters, different solar wind parameters during various periods may drive similar magnetosheath conditions. The main factor driving this variability is the high degree of solar wind‐magnetosheath coupling. In general, the magnetosheath state can be significantly altered during different solar wind conditions. The magnetosheath is also generally asymmetric about the Sun‐Earth line (Dimmock et al., 2017; Walsh et al., 2012), and properties on the dawn and dusk flanks can differ depending on the geometric configuration of the bow shock. Nevertheless, in some studies, a sufficient criteria has been derived to suitably extract magnetosheath data (Chaston et al., 2013). A drawback to this methodology is that the magnetosheath points are not normalized between the boundaries and therefore it is difficult to compare data collected under long intervals containing a variety of driving conditions. However, an advantage of this is that it can be implemented without a solar wind monitor or evaluating boundary models. As a result, this methodology can also be applied to nonterrestrial plasma environments, for example, Volwerk et al. (2016), where upstream monitoring is not continuously available.

## 4 Practical Limitations in Subset Extraction

**Figure 2:**
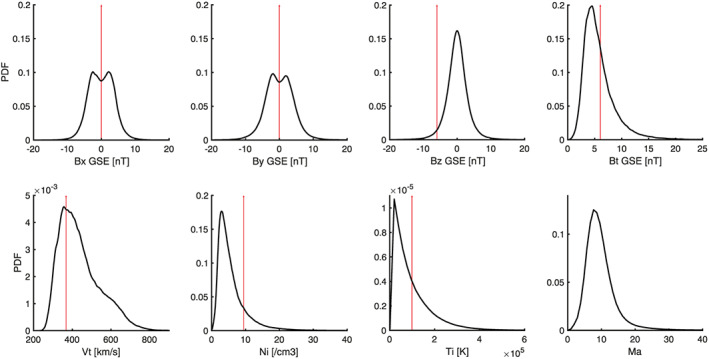
Probability density functions of various solar wind parameters according to the OMNI database from January 2000 to December 2017. The vertical red lines indicate the specific values, which were required for the GEM challenge.

In practice, to investigate solar wind‐magnetosheath coupling, subsets are extracted from the complete statistical database, which correspond to some specific solar wind conditions. This is implemented either to create statistical maps for different upstream states or to match a particular numerical model, which was run under fixed input parameters. However, when extracting subsets, there are several factors to consider, which are worth mentioning. Initially, it is important to consider the general statistical distribution of the solar wind conditions, as this will dictate the quantity of data that is available for any given criterion. The probability distribution functions for various solar wind parameters are shown in Figure 2.

From a statistical standpoint, as shown in Figure 2, it is advisable to avoid very large and extreme values in the tail of the distributions (e.g., *V* > 1,000 km/s), since this will impose severe limitations on the availability of measurements. As a result, when planning a statistical study, the bulk of the distribution should be the focus, as it will provide the largest yield of data points required to perform a meaningful statistical analysis.

The most common IMF orientation is the Parker spiral, noticeable by the two bumps in the*Bx* and*By* PDFs, meaning abundant data should be available for these conditions. The solar wind speed also plays a large role in determining the amount of available data, and speeds between 350 and 500 km/s will yield the most abundant counts. On the other hand, if the purpose of an investigation is to examine stricter and rarer conditions, given a large enough data set, some flexibility can be taken if one does not stray too far from the bulk of the distribution. For conditions well within the tail, such as*Bz*=±10 nT, data will be extremely limited, and it is likely that detailed case studies are a more feasible approach. This also applies to other parameter values that are located in the tail of the PDFs shown in Figure 2. For model runs with similarly rare initial conditions, it is not feasible to statistically compare data with current observational catalogs. Therefore, when creating runs for validation purposes, the occurrence rate of the input conditions should be taken into account.

When extracting subsets using more than one solar wind parameter, the impact on the remaining data can be severe. In such cases, there is no feasible option but to select solar wind conditions, which are the most frequent. Combining more than one criterion will significantly reduce the amount of data. This issue can arise during model‐data comparisons, and therefore, both the model parameters and robustness of the statistical data should be considered simultaneously at the beginning. Nevertheless, some conditions such as a Parker‐spiral IMF and 350 < *V* < 450 km/s solar wind can be applied leaving sufficient data for statistical analysis. Finally, in reality, criterion will always need to be in the form of a range (e.g., 350 < *V* < 450 km/s), and these should be selected individually based on the requirements of the specific study.

## 5 Case Study: GEM Dayside Kinetics Southward IMF Challenge

The geoeffective nature of the solar wind‐magnetosphere interaction during southward*B*_*z*_ has driven the space physics community to advance its understanding of the global system during these conditions. An example of this was the GEM southward IMF challenge (2016–2020) run by the “Dayside Kinetic Processes in Global Solar Wind‐Magnetosphere Interaction” focus group. This challenge was motivated by a case event that provided favorable conjunctions of multiple spacecraft in key locations. Details of this event can be found in the study by Kitamura et al. (2016) and also in other papers within this special issue. Here, we discuss the practicalities of compiling statistical magnetosheath observations for this challenge and in general for specific events. The upstream parameters for this event are listed in Table 1 below. The vertical red arrows in Figure 2 show the location of each of these values in Criteria 0 within the solar wind statistical distributions. It can clearly be seen that*B*_*z*_=−6 nT can be considered to be in the tail of the distribution and is likely to remove significant amounts of data points. The density and temperature can also be considered high, adding to the reduction in available data between October 2007 December 2017. The density of 9.5 cm^−3^ was used for modeling tasks (assuming a pure proton plasma) to better represent the mass density. Upstream measurements indicate that the density is in fact composed of 3–8% alpha particles over the course of the event. Nevertheless, this small deviation is well within the ranges chosen to compile the statistics, and therefore, the solar wind composition will have little to no effect on the results presented here. The remaining conditions are within the reasonable statistical population. In spite of that, it is important to raise a point here, which is that the combination of these parameters will substantially limit the available data. In other words, when multiple solar wind criteria are employed together, the statistical impact will be significant. In fact, to match this condition almost exactly would result in a probability of much less than 0.5%.

**Table 1:** Solar Wind Conditions and Parameter Ranges for the GEM Southward IMF Challenge

#	|**B**|	*B*_*x*_	*B*_*y*_	*B*_*z*_	*V*_*x*_	*V*_*y*_	*V*_*z*_	*N*	*T*_*i*_	Dip tilt
—	[nT]	[nT]	[nT]	[nT]	[km/s]	[km/s]	[km/s]	[cm^−3^]	[eV]	deg
0	6	0	0	−6	−365	0	0	9.5^a^	9	−27°
1	n/a	−2 > 2	−2 > 2	−8 > −4	−390 > −340	n/a	n/a	7.5>12.5	n/a	n/a°
2	n/a	−3 > 3	−3 > 3	−9 > −3	−415 > −315	n/a	n/a	5>15	n/a	n/a
3	n/a	−4 > 4	−4 > 4	−10 > −2	−440 > −290	n/a	n/a	2.5>17.5	n/a	n/a

*Note*. n/a implies that this parameter was not considered

Assuming a pure proton plasma (upstream data indicated 3–8% alpha particles).

**Figure 3:**
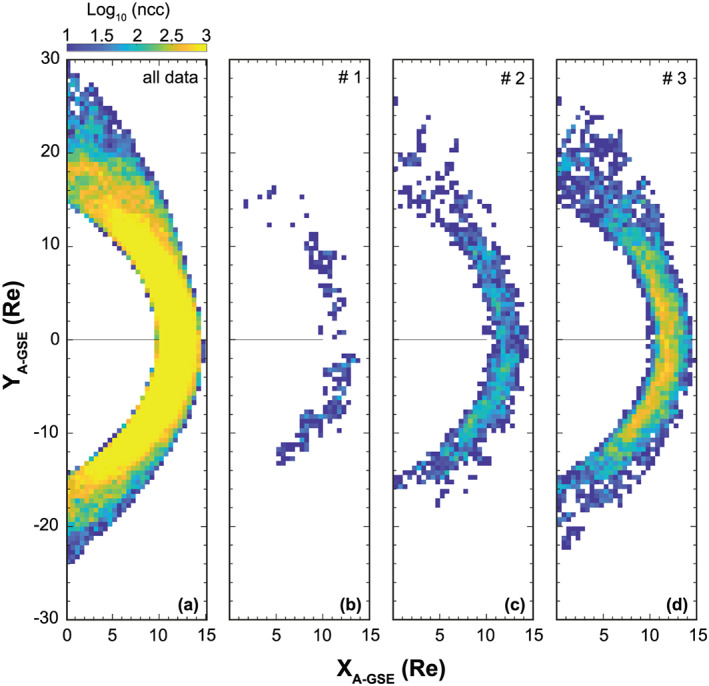
THEMIS dayside magnetosheath coverage for all available data and the conditions specified in Table 1. Each bin is 0.5 × 0.5 Earth radii, and the color indicates the number of data points per bin. It can be seen that the amount of data drastically reduces for the strictest condition and improves as the range is increased around each upstream parameter. This figure highlights the issue of statistical data loss when compiling statistical data for specific upstream conditions.

We have investigated the availability of magnetosheath data for each condition listed in Table 1 based on all available THEMIS data between October 2007 and December 2017 and adopted the model boundary approach described in section 3.1. In practice, it is not feasible to compile statistical data for definite quantities such as Criteria 0, but a range must be applied around each quantity as demonstrated by each of the three criteria (1–3) in Table 1. We investigated the statistical impact of varying degrees of strictness in the parameter limits. This is visualized in Figure 3 by plotting data point counts (ncc) within 0.5 × 0.5 Re bins according to THEMIS measurements between August 2007 and July 2019.

Figure 3a corresponds to all data when no upstream filtering is applied, whereas Figures 3b to Figure 3d are associated with the 1–3 criterion listed in Table 1, respectively. According to Figure 3a, there is abundant data for all conditions with the majority of bins indicating counts above10^4^. Regardless, when attempting to match Specific Condition 0 with relatively narrow ranges imposed on each parameter, the coverage dramatically reduces, and ncc drops to just only10^1^. As the limits around each parameter are gradually loosened, ncc increases significantly. However, as one increases the ranges, it can also be argued that the relevance of these data to the original criterion reduces and the uncertainty increases. As a result, a trade‐off arises between the validity of the statistical data and the data counts in each bin. Therefore, it was the outcome for this GEM challenge that conducting a statistically significant analysis was not feasible. Experience from previous studies and this GEM challenge is that, for the THEMIS data set, a maximum of two upstream limitations should be imposed at a given time. Even in this case, solar wind conditions for an event or model run should lie in the dense region of the probability distribution. The coverage may also change depending on the frame adopted due to the migration of points between bins. For our studies, coverage was assessed individually for each frame, but this effect should be considered when performing coordinate transforms on statistical data. These points should be taken into account for not only future GEM challenges, but other relevant studies. Below we suggest some alternative approaches, which may be used to mitigate some of these issues.

### 5.1 Alternative Southward IMF Criteria

Comprehensive coverage of the magnetosheath does exist when considering the entire statistical data set, as shown in Figure 3a. Despite that, Figure 3b demonstrates that by applying a criterion to several solar wind parameters, the impact on the remaining data set becomes severe. However, if simultaneous limitations are placed on one or two upstream parameters, we will show that it is possible to study the solar wind‐magnetosheath dependency with the required degree of statistical accuracy. In addition, the adopted definition of southward IMF can play a major role. Rather than requiring an exact range of−*Bz*, the strength of−*Bz* can be weighed in relation to|*B*|. This is explained by equation (3) below,

(3) $$B_{z}<-\alpha |B|,$$

where*α* is the scaling factor that dictates the weight of−*Bz* in the IMF vector. The larger the*α* value, the stronger the southward IMF criteria will be, but more data will be removed. Clearly,*α*≈1 is rare, but we will investigate the feasibility of higher values between 0.5 and 0.9.

**Figure 4:**
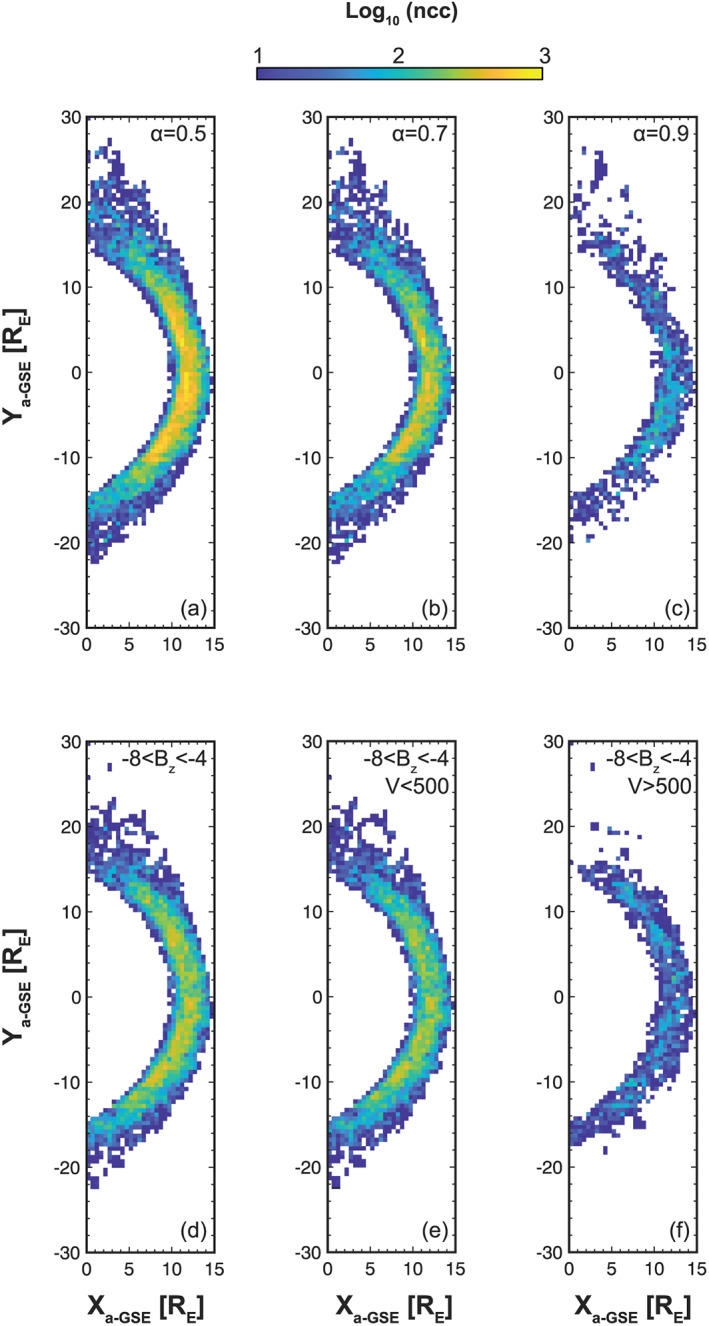
THEMIS dayside magnetosheath coverage for different southward IMF conditions (July 2007 to December 2017).

Figures 4a–4c show the data coverage for*α* = 0.5, 0.7, and 0.9. Figures 4d–4f adopt the−*Bz* limits from Criteria 1 in Table 1 with additional solar wind speed conditions.

From Figure 4a, it is clear that there was a significant amount of data collected during southward IMF, even for the strong case of*α*=0.9. From Figure 4c, around the subsolar region, bins contain approximately 100 points per bin, sufficient for statistical study. Specifically, for the GEM challenge−*Bz* range, data availability is shown in Figure 4d. Interestingly, bin densities remain at sufficient levels of around 1,000 points in many bins. Thus, it is possible to study the magnetosheath under very strong southward IMF, if defined in this manner.

Now we investigate the practicality of more than one upstream parameter limit. Figures 4e and 4f impose two criteria that the solar wind speed is either below and above 500 km/s. Clearly, for lower solar wind speeds, the impact on the data counts is minimal, but the effect for faster speeds is significant. Nevertheless, the counts around the subsolar region in Figure 4f are sufficient, and the coverage is well distributed for investigation focused on this region. If other studies wish to utilize a similar data set and methodology, then our results suggest that only applying criteria to a maximum of two solar wind parameters is advisable in order to maintain good data counts and coverage. In cases where the upstream criteria are too extreme, then an alternative application of this methodology is to identify magnetosheath intervals, which match a given set of conditions. These can then be stored to build a database of events, which can be used for detailed case studies. For a comprehensive description of this methodology, see Dwivedi et al. (2019).

## 6 Applicability of Similar Methodologies to Other Regions

It is worth mentioning that similar methodologies to those discussed here can also be employed to identify other regions, albeit some modifications are needed. Although a detailed analysis of these is beyond the scope of this report, we include them since they will be useful to readers whose interest extends beyond the magnetosheath. Examples of applicable regions are the cusps and the inner‐magnetosphere, two regions of key significance that are also dictated by complex boundaries.

### 6.1 Magnetosphere

In addition to the magnetosheath, a comparable methodology can be adopted for the magnetosphere using the model magnetopause. In this case, a fractional distance describing the normalized proximity to the magnetopause can be defined as

(4) $$F=\frac{\left|\vec{R}\right|}{r_{mp}}.$$

**Figure 5:**
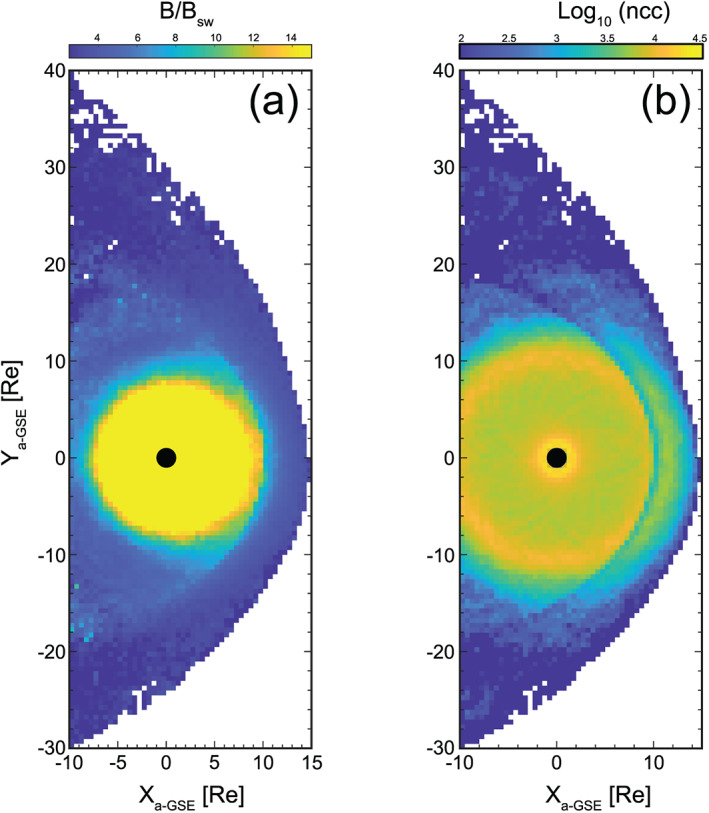
Statistical map of the dayside magnetosphere (January 2008 to December 2017). Panel a shows the normalized magnetic field strength, whereas panel b visualizes the number of points per square 0.5 Re bin.

Figure 5 demonstrates a statistical map of the entire dayside region based on THEMIS data using this approach.

Note that since both regions are binned independently, the discontinuous coverage across the magnetopause in Figure 5b is due to in situ criteria, which remove data in an attempt to eliminate misidentified points close to the boundary (e.g., larger variability). In practice, such criteria can be adjusted based on the nature of the chosen investigation, and this effect can be decreased/increased as a result. In general, when this is implemented, large data points are removed close to boundaries since there is a higher probability of misidentification. In addition, the in situ properties can experience higher variability, resulting in removal of windows (see section 3.1). This should be considered if a challenge wishes to perform statistical studies close to dynamic boundaries.

A notable benefit from mapping the inner‐magnetosphere (using THEMIS) is the increased data counts per bin compared to the magnetosheath. This offers more flexibility when extracting subsets, meaning that studies can investigate solar wind dependencies with additional or stricter criteria while maintaining sufficiently high bin counts. This framework can also be used to study magnetosheath‐magnetosphere coupling by observing the response of both regions to various solar wind conditions. A temporal delay can also be introduced between the solar wind‐magnetosheath‐magnetosphere data to investigate the temporal responses. It is important to point out that although these statistical maps provide an overview of the typical magnetosheath during certain conditions, they are affected by the previous/historical state of the magnetosheath. This may result in a divergence between the model results and these data for purely northward and southward driving conditions, and care has to be taken when interpreting results. Nevertheless, given the appropriate GEM focus group, challenge conditions, and applicability, this methodology and data set may be useful to many future GEM challenges and other studies.

### 6.2 Cusps

**Figure 6:**
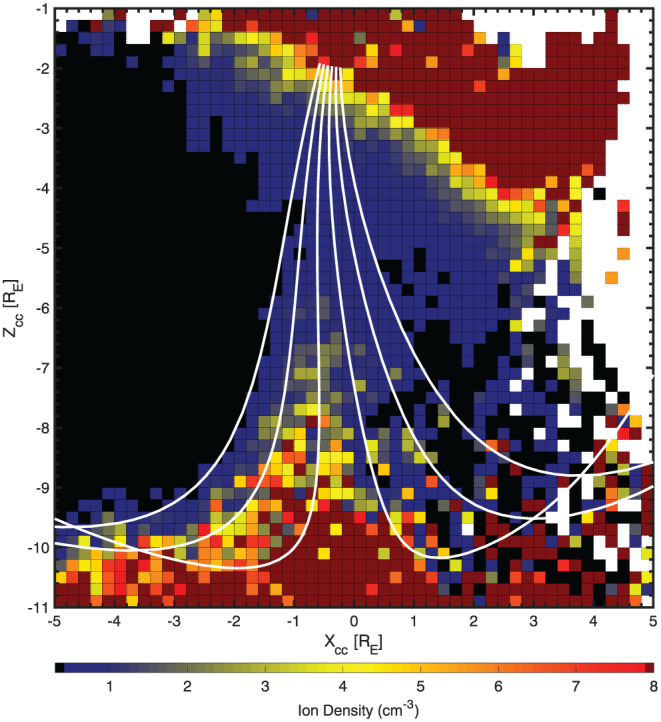
Ion density from Cluster (January 2002 to December 2011) in the southern cusp using a cusp‐centered coordinate system. The white lines show the field lines in the *XZ* plane for typical conditions rotated to a cusp‐centered system to indicate the general location of the cusp.

The geomagnetic cusps are also very dynamic regions, which change in size and shape depending on the prevailing upstream conditions. It is worth mentioning that methodologies addressing this issue have been developed and can be employed in a similar manner to what was discussed above. Similar to the previous regions, the common fundamental problem is to identify the boundaries of the given region for the simultaneous upstream conditions. This can be achieved by employing a magnetospheric field line model such as that implemented by Tsyganenko and Sitnov (2005). The cusp boundaries can then be identified based on the lines on either side of the open field line, or the inner‐most field lines that are orientated tail‐ward and sunward. Such an approach was successfully implemented by Lavraud et al. (2005) using Cluster data. We have also adopted a similar approach; however, data were rotated into a frame in which the *z* axis was aligned with the cusp center. Figure 6 shows a map of the maximum southern cusp density in this frame using over 10 years of Cluster measurements.

The cusp feature is clearly visible along the *z* axis at *x* ∼ 0. The high density toward the top of the graph originates from the plasmasphere but is easily distinguishable from the cusp region. Although not the focus of the current GEM challenge, this cusp frame could be applied to future GEM challenges, which investigate the dependency of cusp field and plasma properties for different upstream criteria.

## 7 Conclusions and Summary

We have discussed the two most commonly adopted methodologies for performing large‐scale statistical analysis of the terrestrial magnetosheath, along with those that apply to different regions. Motivated by the recent dayside kinetic GEM focus group southward IMF challenge, the goal was to provide the practical context on compiling large statistical magnetosheath data sets, which aim to closely match specific upstream solar wind criteria for this, and other events. The aim was to benefit future GEM challenges (and other relevant investigations) with regards to selecting appropriate challenge events or model run parameters to compare with statistical observations. The main conclusions from this work can be described as follows:

1. Multiple methodologies exist, which can be used to compile large statistical observations of the magnetosphere. To some extent, these methods can account for the dynamical shape and position of individual regions.
2. Closely matching several simultaneous upstream parameters will severely restrict the statistical data set, especially when conditions are not in the bulk of the probability distribution.
3. For THEMIS magnetosheath data, similar conditions matching one or two solar wind parameters did produce a data set in which statistically meaningful investigations can be conducted.
4. For southward IMF, rather than requiring strict ranges on*Bx*,*By*, and*Bz* (see Table 1), the strength of*Bz* can be weighed with respect to|*B*| to ensure it is the dominant component. The latter may yield more data points when studying strong southward IMF. Thus, it is important to consider how upstream criteria are defined.
5. If the goal is to compare with statistical data, future GEM challenges and case event studies should carefully select events or model inputs, which yield sufficient statistical observations to make a reasonable comparison. Ideal conditions are Parker‐spiral IMF orientations and/or solar wind velocities between 350 and 500 km/s (see Figure 2).
6. In addition to the magnetosheath, similar methodologies can be adopted to the magnetosphere and cusp regions. Such techniques can also be used to conduct studies of a similar nature.
7. Depending on the region of interest and data set selected, it may be possible to apply additional upstream limitations since the coverage (points per bin) can vary by an order of magnitude between different magnetospheric regions.
8. For many research areas (especially space weather), it is important to understand the physical processes during extreme rare events. However, it will continue to be challenging to relate these to more frequent conditions, which can be studied statistically.

To summarize, careful consideration has to be taken when selecting model input conditions and case study events in which the goal is to compare with large‐scale statistical observations. There are many regions in which statistical data can be compiled (e.g., magnetosheath, magnetosphere, and cusps) using various spacecraft (e.g., THEMIS, Cluster, and MMS). The coverage of each mission for each region should be carefully assessed when selecting events and determining the criteria for extracting subsets. For intense geomagnetic conditions (e.g.,*B*_*z*_<−10 nT and|*V*|>500 km/s), a statistical analysis is difficult due to the lack of data; however, as mentioned in Point 4, modifying the definition of some conditions may help. This will not provide a global coverage but likely yield higher counts in dense areas, which could be enough to perform statistical analysis. If this remains insufficient, then these techniques can be used to search for case events that match rare conditions, which can then be analyzed in detail. Finally, future GEM challenges and similar endeavors should take these matters into consideration from the very start when planning such investigations.
